# Evaluating anthracycline cardiotoxicity associated single nucleotide polymorphisms in a paediatric cohort with early onset cardiomyopathy

**DOI:** 10.1186/s40959-020-00060-0

**Published:** 2020-05-21

**Authors:** Timothy N. McOwan, Lauren A. Craig, Anne Tripdayonis, Kathy Karavendzas, Michael M. Cheung, Enzo R. Porrello, Rachel Conyers, David A. Elliott

**Affiliations:** 1 Murdoch Children’s Research Institute, The Royal Children’s Hospital, Flemington Road, Parkville, Victoria 3052 Australia; 2 Department of Pediatrics, The Royal Children’s Hospital, University of Melbourne, Parkville, Victoria 3052 Australia; 3 Department of Physiology, School of Biomedical Sciences, The University of Melbourne, Parkville, Victoria 3010 Australia; 4 Australian Regenerative Medicine Institute, Monash University, Clayton, Victoria 3800 Australia

**Keywords:** Anthracycline induced cardiomyopathy, Genetic variants, Paediatric cancers

## Abstract

**Background:**

Anthracyclines are a mainstay of chemotherapy. However, a relatively frequent adverse outcome of anthracycline treatment is cardiomyopathy. Multiple genetic studies have begun to dissect the complex genetics underlying cardiac sensitivity to the anthracycline drug class. A number of single nucleotide polymorphisms (SNPs) have been identified to be in linkage disequilibrium with anthracycline induced cardiotoxicity in paediatric populations.

**Methods:**

Here we screened for the presence of SNPs resulting in a missense coding change in a cohort of children with early onset chemotherapy related cardiomyopathy. The SNP identity was evaluated by Sanger sequencing of PCR amplicons from genomic DNA of patients with anthracycline related cardiac dysfunction.

**Results:**

All of the published SNPs were observed within our patient group. There was no correlation between the number of missense variants an individual carried with severity of disease. Furthermore, the time to cardiac disease onset post-treatment was not greater in those individuals carrying a high load of SNPs resulting from missense variants.

**Conclusions:**

We conclude that previously identified missense SNPs are present within a paediatric cohort with early onset heart damage induced by anthracyclines. However, these SNPs require further replication cohorts and functional validation before being deployed to assess anthracycline cardiotoxicity risk in the clinic.

## Background

Anthracyclines exhibit proven efficacy as an anti-neoplastic agent but are associated with significant adverse outcomes, particularly cardiotoxicity [1, 2]. Importantly, the 5-year survival rate for paediatric cancer patients now exceeds 80%, meaning these populations are experiencing higher survival rates and greater life expectancy than ever before [3, 4]. Consequently, the proportion of paediatric cancer patients who survive long enough to develop chemotherapy-related heart damage is on the rise [5, 6]. Hence, late cardiovascular side-effects, and their clinical implications are becoming an increasingly relevant area of research.

Anthracycline cardiotoxicity (ACT) leads to cardiomyocyte damage, ventricular wall thinning and ultimately manifests as a dilated cardiomyopathy [7]. The onset of ACT can be either acute or chronic, and can be clinically appreciable, or subclinical in its presentation. Acute ACT occurs within a week of commencing treatment and occurs in less than 1% of patients [8]. Conversely, chronic ACT is more frequent and develops over an extended period of time in the months to years following treatment [9–11]. Symptomatic ACT is usually defined as congestive cardiac failure, whereas patients with subclinical cardiotoxicity remain asymptomatic. Symptoms and signs of congestive cardiac failure include pulmonary or peripheral oedema, dyspnoea and exercise intolerance [12]. Large cohort studies have found that symptomatic ACT occurs in 1.7–9.8% of childhood cancer survivors [12, 13]. However, the prevalence of subclinical ACT is less certain, and has been reported to occur in 0 to 57% of childhood cancer survivors [14]. The vast disparity in prevalence is partly due to inconsistencies in study populations, particularly varied dose and diagnoses, and is complicated by variability in clinician judgment and assessment criteria. The most widely used criteria for assessing left ventricular subclinical cardiotoxicity is echocardiography, particularly fractional shortening (FS) and ejection fraction (EF). These measures are dependent on preload, afterload and contractility and are a measure of systolic function.

ACT has been associated with a cardiac mortality of 6%, as was found in a 5-year survivorship study of patients diagnosed between the ages of 15 and 39 [15]. Consequently, there is a significant incentive to characterize the risk profile of cancer patients undergoing anthracycline therapy, so as to better guide therapeutic decision-making. So far, proposed predictors include but are not limited to; sex, age at diagnosis, and radiation exposure. Consensus is lacking as to their contribution, if any, to the development of ACT. However, it is generally accepted that cumulative anthracycline dose is the single biggest independent risk factor for ACT [7, 12, 14, 16–19]. As such, the Children’s Oncology Group guidelines for paediatric treatment recommend limiting doses to 450 mg/m^2^ [20]. Further evaluation of patient risk factors has failed to provide a clear consensus regarding clinical characteristics predicting ACT risk. Without knowledge of individual risk, the burden rests on patients, with life-long cardiac monitoring being required.

Nevertheless, personalized follow-up guidelines for cancer survivors are becoming ever more tangible as we enter a forthcoming era of pharmacogenetic-guided treatment. Moving forward, it is hoped we will identify genetic variants that ascribe higher risk for developing ACT, and thus facilitate more effective, individualized risk stratification for paediatric cancer patients. To this end, genetic analysis of large cohorts of cancer patients treated with anthracyclines have verified the existence of many single nucleotide polymorphisms (SNPs) that are associated with the development of ACT [21–31].

Our group recently conducted a retrospective analysis of 481 childhood cancer survivors treated at two tertiary paediatric hospitals. Of the eligible 286 patients, 20 (7.0%) were classified as having severe ACT with FS < 24%. We sought to analyse the frequency of SNPs that have been previously associated with cardiotoxicity in this subset of patients. DNA from this cohort was sequenced, and analyzed for the presence of these SNPs. Their frequencies were then contrasted with that of the general population, with the view to further characterize the risk of ACT in this subset of patients with severe cardiotoxicity.

## Methods

### Patient cohort

This retrospective cohort has been previously reported by our group [32]. The study identified 481 patients from the Haematology-Oncology registry at the Royal Children’s Hospital and Monash Medical Centre, Melbourne, Australia. Patients were eligible to enter the study if they were diagnosed with paediatric cancer between January 2008 and December 2015. Eligibility criteria included a paediatric cancer diagnosis, exposure to anthracycline chemotherapy and at least one echocardiogram performed to assess cardiac function post-chemotherapy. Exclusion criteria included a diagnosis of congenital heart disease. Due to the potential for confounding by acute transient cardiotoxicity, [33] only echocardiograms recorded greater than 17 days after commencing anthracycline therapy were included. Eligible participants were screened for ACT as measured by echocardiogram, specifically fractional shortening and left ventricular ejection fraction (LVEF) using the biplane Simpson’s method. Echocardiogram findings were rated as normal (FS > 28% and without a 10% decline in LVEF from baseline), intermediate (FS 24–28% or a drop in baseline LVEF of > 10%) or severe (FS ≤ 24%). Blood samples were taken and stored for DNA extraction. The total cumulative dose for each patient was determined using doxorubicin equivalents [34]. Ethics approval was given by the institutional review board (HREC25102D).

### DNA sequencing

DNA extraction was performed using QIAgility™ (Qiagen™, Cat #9001532) and QIAmp 96 DNA QIAcube HT kit (Qiagen™, Cat #51331). Primers for PCR amplification and sequencing were designed using Primer-BLAST™ from the National Centre for Biotechnology Information (NCBI). Primers are listed in Supplementary Table 1 (Sigma, Castle Hill, Australia) and amplified using GoTaq® (Promega™, Cat #M30001). DNA products were treated with ExoProStar™ (GE Healthcare Life Sciences, GEHEUS77705). Sequencing was performed by the Australian Genome Research Facility (AGRF) by Sanger sequencing using the amplification primers. Primers and PCR conditions are presented in supplementary methods. Homozygous sequences were confirmed by sub-cloning PCR amplicons into pGEM®-T Easy vector system (Promega, A137A) and sequenced using M13 forward.

## Results

Twenty (20) patients were identified with severe ACT with a fractional shortening < 24% [32]. Of these fifteen consented to genetic analysis to assess the number of anthracycline cardiotoxicity associated SNPs (Table 1). The mean age of these severe ACT patients was 6.2 years at the time of survey with a range of 0 to 17 years (Supplementary Table 2). The mean cumulative anthracycline dose was 211.2 mg/m^2^ (51–435 mg/m^2^). Females are represented disproportionately in this cohort (10 females vs 5 males) (Supplementary Table 2). The distribution of clinical diagnoses is representative of reported frequencies in the paediatric population. Acute lymphoblastic leukaemia (6) and acute myeloid leukaemia (3) were the most common cancers, with a further two each of Wilms Tumour and Ewings Sarcoma and a single individual with Non-Hodgkin’s Lymphoma. Patients received the anthracyclines daunorubicin, doxorubicin, mitoxantrone and idarubicin (Table 1). While the mean time in days from baseline to the first abnormal echocardiogram was 394 days, the range was 17 to 1805 days with a median of 145 days (Table 1). Three of the patients had a significantly delayed time to detection of cardiac systolic dysfunction of greater than 1000 days. Patients 4, 9 and 12 had delay of 1343, 1805 and 1151 days respectively to first abnormal echocardiogram. Excluding this ‘late-onset’ subset, the average days from baseline to the first abnormal echocardiogram was 134 days.

**Table 1:** Individual patient anthracycline dosage, time to first diagnosis and fractional shortening percentage

Individual	Drug^**a**^	Cumulative Dose (mg/m^**2**^)	FS %^**b**^	Time (days) to initial abnormal echo.^**c**^
1	Daun	435	14	276
2	Daun, Mitox	410	23.3	145
3	Dox	356	22.9	267
4	Daun, Dox	325	6	1343
5	Dox	260	23.9	81
6	Dox	255	23.1	64
7	Daun	204	19.7	71
8	Daun	200	21.5	26
9	Daun, Dox	178	23	1805
10	Daun, Dox	175	23.5	253
11	Daun, Ida	161	23.9	17
12	Daun, Dox	147	20.5	1151
13	Daun, Dox	136	19.9	88
14	Dox	72	23.3	214
15	Dox	51	23.9	106

^**a**^*Daun* daunorubicin; *Dox* doxorubin; *Mitox* mitroxantrone; *Ida* Idarubicin. ^**b**^ most abnormal record of fractional shortening. ^**c**^*echo* echocardiography

Previously identified SNPs associated with anthracycline-induced cardiotoxicity are summarised in Supplementary Table 3. These variants are distributed between missense, synonymous, intronic, and upstream and downstream of associated genes. The frequency of each variant in the 1000 Genomes (1KGP) and Genomes Aggregate (gnomAD) databases is presented. These frequencies range from 4 to 82%. Several of these genes have been previously identified in association with other diseases [35, 36]. Of the variants previously identified, missense variants are reported in eight genes. These eight genes were the focus of our study, as they were considered most likely to directly affect the mechanism of cardiotoxicity. The frequency of these genetic variants in our cohort was compared to the global frequencies reported in the 1000 Genomes Project.

Patients with severe ACT were then tested for the presence of eight previously-reported missense variants, Table 2. All of these variants were found in at least one patient, however, none were found in all patients. Variants in *ETFB*, *ABCC2*, *GPR35* and *RARG* were reported as heterozygous variants, while *CYBA*, *NOS3* and *CBR3* were reported as both homozygous and heterozygous variants. The highest burden of variants in a single patient was 7, in a combination of both homozygous and heterozygous variants. One patient reported none of the eight-missense variants screened. The variant burden in our cohort of patients with severe ACT ranged from 0 to 7. While this is an observational study evaluating the reported SNPs in a small cohort of children treated with anthracycline the frequency of the variants observed is broadly in line with the known frequency of these SNPs in the general population (Table 3). Total cumulative dose of anthracycline given did not correlate with the number of variants reported. Similarly, the number of variants each patient carries did not relate to the time from baseline to first abnormal echocardiogram or the most abnormal FS.

**Table 2:** Presence and zygosity status of anthracycline cardiotoxicity associated SNPs in patients with cardiotoxicity

	***NOS3***	***ETFB***	***CBR3***	***ABCC2 1***	***ABCC2 2***	***RARG***	***GRP35***	***CYBA***
	rs1799983	rs79338777	rs1056892	rs17222723	rs8187710	rs2229774	rs12468485	rs4673
Patient								
1	Het^b^							
2		Het	Hom	Het	Het			
3	Hom^a^			Het	Het			
4	Hom	Het	Het	Het	Het	Het		Het
5	Het		Het	Het	Het			Het
6	Het		Hom			Het		Hom
7								
8							Het	Hom
9	Hom		Hom					Het
10	Hom			Het	Het	Het		Het
11	Hom		Het					Het
12	Hom		Het					Het
13	Hom		Hom				Het	Hom
14	Hom		Het	Het	Het			Het
15	Het							

^a^*Het* heterozygous; ^b^*Hom* homozygous

**Table 3:** Frequency of missense variants in patients with severe anthracycline-induced cardiotoxicity compared to global population

Gene	*NOS3*	*ETFB*	*CBR3*	*ABCC2 1*	*ABCC2 2*	*RARG*	*GRP35*	*CYBA*
SNP	rs1799983	rs79338777	rs1056892	rs17222723	rs8187710	rs2229774	rs12468485	rs4673
Total (of 15)	12	2	9	6	6	3	2	10
Percentage	80	13.33	60	40	40	20	13.33	66.67
Reported Frequency^a^	82	8	43	4	7	9	5	66

^a^ 1000 genomes reported global frequency

## Discussion

In this study we have examined genetic variants previously associated with ACT in a cohort of severely affected paediatric patients. Severe ACT was defined as a fractional shortening < 24% as measured by echocardiogram, which was observed in 7% of the retrospective cohort [32]. These patients varied in clinical diagnosis, age, total cumulative anthracycline dose and time to abnormal echocardiogram, reflecting the patient population treated at two major Australian paediatric hospitals. Our data suggest that, at present these variants cannot accurately predict cardiac sensitivity to anthracycline.

We focussed on the previously reported non-synonomous variants associated with ACT, reasoning that these protein changes were most likely to directly alter anthracycline pharmacodynamics, thus contributing to cardiotoxicity [37].. A total of 15 genes have been associated with a genetic predisposition to anthracycline cardiomyopathy [21–27, 30, 38–40]. We excluded screening for variants in non-coding sequences or those that encoded a synonymous amino acid change, focussing on variants which modified the amino acid sequence of a given protein. For example, *RARG* rs2229774 (*RARG*^*c.1280C > T*^; p.S427L) was significantly associated with ACT in a cohort of 280 European Canadian patients (32 ACT cases and 248 controls) and a replication cohort of 96 European Dutch (22 cases and 74 controls) [21]. In an attempt to validate these associations in the setting of early onset severe ACT, our study identified that 14 out of 15 patients had at least one of the missense variants associated with cardiotoxicity to date. Nevertheless, incorporating these genetic markers into practice requires replication studies and further functional validation [41].

Importantly, both the onset of cardiotoxicity after anthracycline treatment and the dose at which cardiotoxicity was observed did not correlate with the burden of variants within this cohort. While all variants examined where found within the cohort, with 14 out of 15 patients having at least one variant, no single variant was observed in all patients. In agreement with our current understanding, this suggests the genetic risk of ACT is polygenic in nature and supports further genetic studies with larger cohorts.

## Conclusion

While previously published missense variants are found within our ACT cohort, further functional validation is required to determine the pathogenic mechanism underlying ACT in individuals carrying these variants. The clinical utility of these variants as predictors of anthracycline cardiotoxicity requires detailed studies in much larger cohorts of paediatric cancer survivors.

## Supplementary information

**Additional file 1: Table S1.** Primers amplifying DNA including the identified SNP for identification of previously identified missense variants associated with anthracycline cardiotoxicity. **Table S2.** Sex, age at diagnosis and tumour type of study cohort*. **Table S3.** Published single nucleotide polymorphisms (SNPs) associated with anthracycline-induced cardiotoxicity

## Data Availability

All data and materials are available upon request.

## Abbreviations

SNP: Single nucleotide polymorphism
ACT: Anthracycline induced cardiotoxicity
LVEF: Left ventricular ejection fraction
FS: Fractional shortening
