# Supplementary material for: Evaluating anthracycline cardiotoxicity associated single nucleotide polymorphisms in a paediatric cohort with early onset cardiomyopathy
Source: Cardiooncology. 2020 May 21;6:5. doi: 10.1186/s40959-020-00060-0 (PMC7243302; doi:10.1186/s40959-020-00060-0)
Supplement: Supplementary file 1 — Additional file 1: Table S1. Primers amplifying DNA including the identified SNP for identification of previously identified missense variants associated with anthracycline cardiotoxicity. Table S2. Sex, age at diagnosis and tumour type of study cohort*. Table S3. Published single nucleotide polymorphisms (SNPs) associated with anthracycline-induced cardiotoxicity [file 40959_2020_60_MOESM1_ESM.docx]

Supplementary Table 1. Primers amplifying DNA including the identified SNP for identification of previously identified missense variants associated with anthracycline cardiotoxicity.

| **Gene** | **Variant location** | **Forward Primer** | **Reverse Primer** |
| --- | --- | --- | --- |
|  |  |  |  |
| *CELF4* | chr18:35077028 | TAGCAACTGTGACTGGGACG | TTCTCTCTTGGTGGGCTCTG |
| *HAS3* | chr16:69143577 | GCTACCAGTTCATCCACACG | AGGTCAGGGAAGGAGATGC |
| *ABCC1* | chr16:16235681 | CCTACTGCCTCGGATCTCTC | CTGGCCCAAAACGAGAAC |
| *ETFB* | Chr19: 51857738 | CAGGCTTCACTCGGATCTG | GAGGAGCAGTGACAACAGTAGG |
| *ABCC5* | chr3:183737356 | CCATTTCAGGTAGATCAAACGC | GAGTGGCTGCTCAAGTTTCC |
| *CAT* | chr11:34460231 | CTTCTGATTGGCTGCTCGG | ATTCCGTCTGCAAAACTGGC |
|  | chr11:34460704 | CTGCAGTGTTCTGCACAGC | CCCTCAATCTGTCCTCAAGC |
| *NOS3* | chr7:150696111 | CAGGAGACAGTGGATGGAGG | TGCAGGCCCTTCTTGAGAG |
| *RARG* | chr12:53605545 | GTGCCTCTGTCCTCCTGAGC | CTCATTGGAAGGGGTGGG |
| *GPR35* | chr2:241570127 | GGTCTTCTGCTCCCTGAAGG | GGAACTCCTTGGCCATGTAG |
| *SLC28A3* | chr9:86900926 | AAGGTGGGTGGGAAGTTGG | GCACCAATGGTGTCCATCC |
| *NCF4* | chr22:37256846 | GCCTGGGGAAGAGTTTGG | GGCCCACACTTCCTCTTACC |
| *RAC2* | chr22:37632770 | CCCAATTCAGAAAGGCCCAC | CCATTGCCCTGAGAACCAAG |
| *CBR3* | chr21:37518706 | TGAGGTGCATGAGAGGGAAG | CCGAAGCAGACGTTTACCAG |
| *ABCC2* | chr10:101595996 | TTATGTGTCTACCTCCCGCC | CCCTCCCACCGCTAATATC |
|  | chr10:101611294 | AAATGCCTAGACTTGAGATGCTGC | CTAACCCATGGGGCCTTCTG |
| *CYBA* | chr16:88713236 | GGTGGAGCTTGGTTTCTCAC | GGACCCGAATTTTTGTTTGG |
| *UGT1A6* | chr2:234602277 | CTCTTTTCACAGACCCAGC | ACAGCCAAACAGAGACCTTC |

**Supplementary Table 2.** Sex, age at diagnosis and tumour type of study cohort*.

| **Sex** | **Age at Diagnosis**  **(Years)** | **Tumour** |
| --- | --- | --- |
| F | 0 | AML ^a^ |
| F | 1 | ALL ^b^ |
| F | 2 | NHL ^c^ |
| F | 3 | AML |
| F | 3 | WT ^d^ |
| M | 3 | ALL |
| F | 5 | ALL |
| F | 6 | ALL |
| M | 8 | AML |
| F | 8 | ES ^e^ |
| M | 8 | AML |
| M | 10 | WT |
| M | 12 | ALL ^d^ |
| F | 15 | ALL |
| F | 17 | ES |

^a^ AML = Acute Myeloid Leukaemia; ^b^ ALL = Acute Lymphoblastic Leukaemia; ^c^ Non-Hodgkin’s Lymphoma;^d^ Wilms’ Tumour; ^e^ Ewing’s Sarcoma.

*Data for each individual in the study can be requested from the authors.

**Supplementary Table 3.** Published single nucleotide polymorphisms (SNPs) associated with anthracycline-induced cardiotoxicity

| **Gene** | **Variant location** | **Variant Type** | **Amino acid change** | **rs ID** | **gnomAD**  **Variant**  **Frequency** | **Global Variant Frequency*** | **Author/Year** |
| --- | --- | --- | --- | --- | --- | --- | --- |
| *ETFB* | chr19: 51857738 | Missense | p.Pro52Leu | rs79338777 | 0.07269 | 8% | Ruiz-Pinto et al. 2017 |
| *SOD2* | chr6:160113872 | Missense | p.Val16Ala | rs4880 | 0.4834 | 41% | Rajic et al. 2009 |
| *NOS3* | chr7:150696111 | Missense | p.Asp298Glu | rs1799983 | 0.7514 | 82% | Krajinovic et al. 2015 |
| *RARG* | chr12:53605545 | Missense | p.Ser427Leu | rs2229774 | 0.07327 | 9% | Aminkeng et al. 2005 |
| *GPR35* | chr2:241570127 | Missense | p.Thr253Met | rs12468485 | 0.06074 | 5% | Ruiz-Pinto et al. 2017 |
| *CBR3* | chr21:37518706 | Missense | p.Val244Met | rs1056892 | 0.3688 | 43% | Blanco et al. 2008 |
| *ABCC2* | chr10:101595996 | Missense | p.Val1188Glu | rs17222723 | 0.04541 | 4% | Wojnowski et al. 2005 |
|  | chr10:101611294 | Missense | p.Cys1515Tyr | rs8187710 | 0.05487 | 7% | Wojnowski et al. 2005 |
| *CYBA* | chr16:88713236 | Missense | p.Tyr72His | rs4673 | 0.694 | 66% | Visscher et al. 2012 |
| *HAS3* | chr16:69143577 | Synonymous | p.Ala93Ala | rs2232228 | 0.3942 | 34% | Wang et al. 2014 |
| *SLC28A3* | chr9:86900926 | Synonymous | p.Leu461Leu | rs7853758 | 0.156 | 20% | Visscher et al. 2012 |
| *UGT1A6* | chr2:234602277 | Synonymous | p.Val209Val | rs17863783 | 0.03966 | 7% | Visscher et al. 2013 |
| *CELF4* | chr18:35077028 | Intronic |  | rs1786814 | 0.1542 | 13% | Wang et al. 2016 |
| *CAT* | chr11:34460704 | Intronic |  | rs10836235 | 0.09973 | 13% | Rajic et al. 2009 |
| *RAC2* | chr22:37632770 | Intronic |  | rs13058338 | 0.1957 | 16% | Wojnowski et al. 2005 |
| *ABCC5* | chr3:183737356 | Upstream |  | rs7627754 | 0.2009 | 26% | Krajinovic et al. 2015 |
| *CAT* | chr11:34460231 | Upstream |  | rs1001179 | 0.1631 | 13% | Rajic et al. 2009 |
| *NCF4* | chr22:37256846 | 5' Region |  | rs1883112 | 0.4149 | 41% | Wojnowski et al. 2005 |
| *ABCC1* | chr16:16235681 | 3' UTR variant | | rs3743527 | 0.2268 | 29% | Semsei et al. 2012 |

* Global Variant Frequency from 1000 Genomes.
